# Factors influencing downstaging after neoadjuvant long-course chemoradiotherapy in rectal carcinoma

**DOI:** 10.1007/s00384-022-04174-y

**Published:** 2022-05-11

**Authors:** Valerie K. B. Kohl, Klaus Weber, Maximilian Brunner, Carol I. Geppert, Rainer Fietkau, Robert Grützmann, Sabine Semrau, Susanne Merkel

**Affiliations:** 1 Department of Surgery, Friedrich-Alexander-Universität Erlangen-Nürnberg, Erlangen, Germany; 2 Comprehensive Cancer Center Erlangen–European Metropolitan Area of Nuremberg (CCC ER-EMN), Erlangen, Germany; 3 Institute of Pathology, Friedrich-Alexander-Universität Erlangen-Nürnberg, Erlangen, Germany; 4 Department of Radiation Oncology, Friedrich-Alexander-Universität Erlangen-Nürnberg, Erlangen, Germany; 5 Department of Surgery, University Hospital Erlangen, Krankenhausstr. 12, 91054 Erlangen, Germany

**Keywords:** Rectal carcinoma, Neoadjuvant chemoradiotherapy, Downstaging, Tumour regression, Prediction of pathological response, Prognostic factors

## Abstract

**Purpose:**

This single-centre cohort study was designed to identify factors that can predict primary tumour downstaging by neoadjuvant chemoradiotherapy (nCRT) in rectal carcinoma.

**Methods:**

Prospectively collected data from 555 patients with clinical T category (cT) cT3-4 rectal carcinoma treated between 1995 and 2019 were retrospectively analysed. All patients received long-term neoadjuvant chemoradiotherapy followed by surgery with curative intent at the Department of Surgery, University Hospital Erlangen, Germany. Patient-, tumour- and treatment-related factors with a potential impact on the downstaging of rectal carcinoma to pathological T category (pT) ≤ ypT2 and ypT0 were analysed in univariate and multivariate logistic regression analyses. The prognosis of patients with and without downstaging of the primary tumour was compared.

**Results:**

A total of 288 (51.9%) patients showed downstaging to ≤ ypT2. Eighty-six (15.5%) patients achieved clinical complete regression (ypT0). In the multivariate logistic regression analysis, the factors cT category, BMI, ECOG score, CEA, histological type, extension in the rectum and year of the start of treatment were found to be independent factors for predicting downstaging to ≤ ypT2 after neoadjuvant chemoradiotherapy. The year of treatment initiation also remained an independent significant predictor for pathological complete regression. The prognosis was superior in patients with downstaging to ≤ ypT2 in terms of locoregional and distant recurrence as well as disease-free and overall survival.

**Conclusion:**

Factors predicting downstaging after long-term nCRT could be identified. This may be helpful for counselling patients and selecting the optimal treatment for patients with advanced rectal carcinoma.

**Supplementary information:**

The online version contains supplementary material available at 10.1007/s00384-022-04174-y.

## Introduction

Colorectal cancer is one of the most common cancers worldwide and was responsible for nearly 10% of cancer deaths in 2020 [1]. In recent decades, prognosis has been significantly improved by two important factors: the total mesorectal excision (TME) technique and radio(chemo)therapy for patients with advanced rectal carcinoma. As a result of both, a significant reduction in locoregional recurrence rates has been observed.

The concept of total mesorectal excision (TME) for rectal cancer was introduced by R. J. Heald [2]. With this technique, potential tumour deposits in lymph nodes and tumour cells within the mesorectum are completely removed en bloc with the tumour. The risk of local tumour recurrence can thus be lowered considerably [2–4].

In the multimodal therapy of advanced rectal cancer, neoadjuvant radiation represents an important component of the treatment. In short-course radiotherapy, patients are irradiated with 5 Gy for 5 consecutive days followed by surgery within 1 week after completion. In neoadjuvant long-term chemoradiotherapy (nCRT), radiation is administered for 6 weeks with concomitant chemotherapy during the first and fifth week, and surgery is performed six to eight weeks after the end of radiation. During this period, regression of the tumour can be expected. The CAO/ARO/AIO-94 trial has shown that preoperative chemoradiotherapy leads to better local control and is associated with lower toxicity than postoperative chemoradiotherapy [5–7]. Since then, neoadjuvant chemoradiotherapy followed by TME has become the standard treatment for locally advanced rectal cancer (cT3-4 or cN +) in many countries, including Germany.

The success of neoadjuvant therapy is mainly described in the histopathological examination of the resected specimen with downstaging from the clinical pretherapeutic cT category to the pathological ypT category after chemoradiation. Due to the lower sensitivity and specificity of the clinical N category, the corresponding downstaging of the regional lymph node status from cN + to ypN0 is less appropriate to describe this success. Furthermore, a reduction in tumour cells can be described and classified according to regression systems such as that of Dworak [8].

Neoadjuvant long-term chemoradiotherapy, including the subsequent consolidation phase, lasts at least 12 weeks until the carcinoma is finally removed. It is therefore important to know which patients will particularly benefit from this treatment. The aim of this study was to investigate which patient-, tumour- and treatment-related factors have an impact on the downstaging of rectal carcinoma. The primary endpoint of this study was downstaging from cT3-4 to a ypT category ≤ ypT2, and the secondary endpoint was downstaging of rectal carcinoma to ypT0 (pathological complete regression).

## Methods

This single-centre cohort study included a total of 567 patients with primary rectal carcinoma (cT3-4 any cN M0) who underwent neoadjuvant long-course chemoradiotherapy (nCRT) followed by surgery with curative intent (R0/R1) at the Department of Surgery, University Hospital Erlangen, Germany, between 1995 and 2019. Patients were selected based on the following inclusion criteria: solitary invasive rectal carcinoma with a distal margin < 12 cm from the anal verge, measured with a rigid sigmoidoscope; carcinoma not associated with familial polyposis, ulcerative colitis or Crohn´s disease; and pretherapeutic staging cT3-4 any cN M0. Patients with other synchronous or metachronous malignancies and patients with clinical (nearly) complete remission on a ‘watch and wait’ strategy after nCRT were excluded. One patient with a rectal perforation 10 days after nCRT and therefore premature tumour resection was excluded, as were 11 patients with delayed resection after an extended interval between the end of nCRT and surgery of more than 6 months. Overall, 555 patients were analysed.

General epidemiological data, clinical findings and treatment as well as histopathological findings were collected prospectively at the Erlangen Registry for Colorectal Carcinomas (ERCRC). Retrospectively, additional potential predictive factors for downstaging were assessed.

Prior to the start of nCRT, all patients underwent thorough preoperative diagnostics and staging. In accordance with the respective current German S3 guideline [9], the following examinations were performed: rectoscopy with biopsy, abdominopelvic computed tomography (CT) and/or, from 2005, increasingly magnetic resonance imaging (MRI) to assess the depth of tumour invasion, lymph node status and involvement of the mesorectal fascia (MRF), chest X-ray/CT and serum tumour markers (CEA, CA19-9).

All patients underwent long-course nCRT. Radiotherapy was given to a cumulative dose of 50.4 Gy (28 fractions of 1.8 Gy, 5 days per week) to the pelvis. Chemotherapy was given either in the first and fifth week with infusional 5-FU ^1000 mg/m2^ d1-5 or 5-FU ^250 mg/m2^ d1-14,21–34 plus oxaliplatin ^50 mg/m2^ d1,8,21,29. In a few patients, oxaliplatin was replaced by irinotecan or irinotecan monotherapy. The selection of chemotherapeutic agents depended on whether the patients were treated at the centre, at their local hospital or within clinical trials. Ninety-six of the patients received hyperthermia in an ongoing study. This involved heating the tumour tissue to 40 to 43°C for approximately an hour, once or twice a week for up to 10 sessions.

Six to eight weeks after completion of chemoradiotherapy, TME surgery was performed with precise dissection within the visceral and parietal fascia sparing the autonomic nerve structures whenever possible and appropriate. The final decision on the surgical method, in particular sphincter preservation yes or no, was always made intraoperatively.

The detailed documentation of the histopathological findings enabled the classification of the carcinomas according to the 8th edition of the TNM classification of the UICC [10]. A tumour that has invaded the perirectal fat tissue is classified as T3, and a tumour with perforation of the visceral peritoneum or invasion into other organs or structures is classified as T4.

Tumour downstaging was defined according to Yoon [11] as histopathologic downstaging of the primary tumour from cT3-4 to ypT2 or less. The Dworak grading system with a scale from 0 to 4 was used to determine histopathological tumour regression [8]. Grade 4 was defined as pathological complete regression corresponding to ypT0. Pathological complete regression (pCR) is the absolute absence of tumour cells in the resected specimen.

Body mass index (BMI) was classified according to the definition of the World Health Organization (WHO): underweight (BMI < 18.5 kg/m^2^), normal weight (BMI 18.5–24.9 kg/m^2^), overweight (BMI 25.0–29.9 kg/m^2^) and obese (BMI ≥ 30.0 kg/m^2^) [12].

Patients were followed up for at least 5 years, in the first 2 years at 3-month intervals and thereafter at 6-month intervals. In 2004, the follow-up interval was changed according to the guidelines: every 6 months for the first 2 years and annually thereafter. Follow-up data were collected either through follow-up visits at the university hospital or through written correspondence with the patients’ treating physicians. After 5 years, at least a vital status check was carried out annually at the local registration office.

### Statistical analysis

To compare categorical data, the χ2 test was used, and Fisher’s exact test was used for values < 5. The Mann–Whitney U test was applied for quantitative data. Univariate logistic regression analyses were used to assess the interaction of potential predictive factors on downstaging of the T category. Variables that reached significance at *p* < 0.05 in the univariate analysis were included in a multivariate model. As the distance of the carcinoma to the mesorectal fascia (MRF) was determined in less than 50% of the patients (*n* = 259), this factor was not included in the multivariate analysis.

For the analysis of prognosis, only patients treated between 1995 and 2015 with a potential follow-up of at least 5 years were considered. Two patients with missing follow-up information were excluded; thus, 471 patients were evaluated. Disease-free survival (DFS) was defined as the time interval from primary surgery to the occurrence of local recurrence, distant metastasis or death by any cause. For overall survival (OS), the patient’s death from any cause was determined as the endpoint. Survival was estimated using the Kaplan–Meier method, and survival curves were compared using the log-rank test. The 95% confidence intervals (95% CI) were calculated according to Greenwood [13]. A two-sided *p* value < 0.05 indicated a significant difference. All analyses were performed with the statistical software IBM SPSS® version 28 (IBM, Armonk, NY, USA).

## Results

The patients’ tumour and treatment characteristics are summarised in Table 1. At a median of 7.3 weeks (IQR 6.7–8.6) after the completion of neoadjuvant chemoradiotherapy, TME surgery was performed.

**Table 1:** Tumour and treatment characteristics of 555 patients treated with neoadjuvant chemoradiotherapy

	***n***	**%**
Age median (range) (years)	62.0 (24–86)	
Sex		
Male	398	71.7
Female	157	28.3
BMI (kg/cm^2^)		
< 18.5	13	2.3
18.5–24.9	197	35.5
25.0–29.9	222	40.0
≥ 30.0	123	22.2
ECOG score		
0–1	478	93.2
2–3	35	6.8
Histologic type		
Adenocarcinoma	528	95.1
Mucinous adenocarcinoma	24	4.3
Signet ring cell carcinoma	1	0.2
Medullary carcinoma	2	0.4
Distance from anal verge		
< 6 cm	286	51.5
6–<12 cm	269	48.5
Distance from mesorectal fascia (MRI)		
> 1 mm	53	20.5
≤ 1 mm	206	79.5
Stoma required before nCRT	43	7.7
Clinical T category		
cT3	441	79.5
cT4	114	20.5
Clinical N category		
cN0	135	24.3
cN +	416	75.7
Chemotherapy regimen		
5-FU or Capecitabine	243	44.2
Oxaliplatin-based	291	52.9
Irinotecan-based	16	2.9
Hyperthermia		
Yes	95	17.2
No	457	82.8

*BMI* body mass index, *ECOG* Eastern Cooperative Oncology Group, *MRI* magnetic resonance imaging, *nCRT* neoadjuvant chemoradiotherapy, *5-FU* 5-fluorouracil

The distribution of pathologic tumour response in terms of ypT category and Dworak regression grade is illustrated in Table 2. A total of 288 (51.9%) patients presented with downstaging to ypT2 or less after neoadjuvant chemoradiotherapy. Eighty-six (15.5%) were classified as ypT0 as pathological complete regression (Dworak grade 4).

**Table 2:** Pathological tumour response after nCRT

**ypT category**		**Dworak primary tumour regression grade**					
		**Grade 4 (100%)**	**Grade 3 (> 50– < 100%)**	**Grade 2 (> 25–50%)**	**Grade 1 (1–25%)**	**Grade 0 (no regression)**	**Unknown**
	***n* (%)**	***n* (%)**	***n* (%)**	***n* (%)**	***n* (%)**	***n* (%)**	***n* (%)**
ypT0	86 (15.5)	86 (100.0)	0	0	0	0	0
ypT1	29 (5.2)	0	20 (69.0)	5 (17.2)	3 (10.3)	1 (3.5)	0
ypT2	173 (31.2)	0	122 (70.5)	31 (17.9)	12 (6.9)	6 (3.5)	2 (1.2)
ypT3	238 (42.9)	0	141 (59.2)	62 (26.1)	29 (12.2)	5 (2.1)	1 (0.4)
ypT4	29 (5.2)	0	12 (41.4)	6 (20.7)	8 (27.6)	1 (3.4)	2 (6.9)
	555 (100.0)	86 (15.5)	295 (53.2)	104 (18.7)	52 (9.4)	13 (2.3)	5 (0.9)

Patient-related predictive factors associated with a significantly higher frequency of downstaging to ≤ ypT2 were as follows: a favourable ECOG performance score 0–1 (54% vs 20%; *p* < 0.001), a regular CEA level < 5 ng/ml (58% vs 35%; *p* < 0.001), a low CRP value < 5 mg/l (60% vs 35–52%; *p* = 0.018) and a normal weight or overweight body mass index (BMI) (55–58% vs 23–40%; *p* = 0.002, Table 3).

**Table 3:** Potential patient-related predictors of downstaging (*n* = 555)

Characteristics	≤ ypT2	ypT3-4	*p*	ypT0	ypT1-4	*p*
	*n* (%)	*n* (%)		*n* (%)	*n* (%)	
	288 (51.9)	267 (48.1)		86 (15.5)	469 (84.5)	
Age (years)						
≤ 60	135 (53.8)	116 (46.2)		42 (16.7)	209 (83.3)	
> 60	153 (50.3)	151 (49.7)	0.358	44 (14.5)	260 (85.5)	0.464
Sex						
Male	212 (53.3)	186 (46.7)		68 (17.1)	330 (82.9)	
Female	76 (48.4)	81 (51.6)	0.302	18 (11.5)	139 (88.5)	0.099
BMI (kg/m^2^)						
< 18.5	3 (23.1)	10 (76.9)		1 (7.7)	12 (92.3)	
18.5–24.9	114 (57.9)	83 (42.1)		30 (15.2)	167 (84.8)	
25.0–29.9	122 (55.0)	100 (45.0)		39 (17.6)	183 (82.4)	
≥ 30.0	49 (39.8)	74 (60.2)	**0.002**	16 (13.0)	107 (81.0)	0.657
ECOG score*						
0–1	260 (54.4)	218 (45.6)		80 (16.7)	398 (83.3)	
2–3	7 (20.0)	28 (80.0)	** < 0.001**	2 (5.7)	33 (94.3)	0.097
CEA (ng/ml)*						
< 5	203 (57.5)	150 (42.5)		61 (17.3)	292 (82.7)	
≥ 5	51 (35.4)	93 (64.6)	** < 0.001**	16 (11.1)	128 (88.9)	0.085
CRP (mg/l)*						
Norm < 5	140 (59.8)	94 (40.2)		44 (18.8)	190 (81.2)	
5– < 10	50 (51.5)	47 (48.5)		18 (18.6)	79 (81.4)	
10– < 50	37 (43.5)	48 (56.5)		6 (7.1)	79 (92.9)	
≥ 50	7 (35.0)	13 (65.0)	**0.018**	3 (15.0)	17 (85.0)	0.055

*BMI* body mass index, *ECOG* Eastern Cooperative Oncology Group, *CEA* carcinoembryonic antigen, *CRP* C reactive protein

*Missing values: ECOG score *n* = 42, CEA *n* = 42, CRP *n* = 119

Tumour-related predictive factors and their association with downstaging are presented in Table 4. Downstaging was observed significantly more frequently in carcinomas with the following characteristics: cT3 (56% vs 36%; *p* < 0.001), insular or semicircular extension in the rectum (55–65% vs 43%; *p* < 0.001), adenocarcinoma as the histological type (53% vs 22%; *p* = 0.002) and distance to mesorectal fascia (MRF) > 1 mm (64% vs 48%; *p* = 0.037).

**Table 4:** Potential tumour-related predictors of downstaging (*n* = 555)

Characteristics	≤ ypT2	ypT3-4	*p*	ypT0	ypT1-4	*p*
	*n* (%)	*n* (%)		*n* (%)	*n* (%)	
	288 (51.9)	267 (48.1)		86 (15.5)	469 (84.5)	
Clinical T category						
cT3	247 (56.0)	194 (44.0)		73 (16.6)	368 (83.4)	
cT4	41 (36.0)	73 (64.0)	** < 0.001**	13 (11.4)	101 (88.6)	0.176
Clinical N category*						
cN0	74 (54.8)	61 (45.2)		20 (14.8)	115 (85.2)	
cN +	214 (51.4)	202 (48.6)	0.495	66 (15.9)	350 (84.1)	0.770
Distance from anal verge						
< 6 cm	149 (52.1)	137 (47.9)		50 (17.25	236 (82.5)	
6–<12 cm	139 (51.7)	130 (48.3)	0.920	36 (13.4)	233 (86.6)	0.182
EVI (CT/MRI)*						
Negative	239 (53.3)	209 (46.7)		3 (10.3)	26 (89.7)	
Positive	12 (41.4)	17 (58.6)	0.211	75 (16.7)	373 (83.3)	0.602
Distance from MRF in MRI*						
> 1 mm	34 (64.2)	19 (35.8)		13 (24.5)	40 (75.5)	
≤ 1 mm	99 (48.1)	107 (51.9)	**0.037**	34 (16.5)	172 (83.5)	0.176
Ulceration*						
No	134 (53.8)	115 (46.2)		35 (14.1)	214 (85.9)	
Yes	124 (53.9)	106 (46.1)	0.983	43 (18.7)	187 (81.3)	0.169
Extension in the rectum (clinical)*						
Insular	106 (64.6)	58 (35.4)		28 (17.1)	136 (82.9)	
Semicircular	72 (55.0)	59 (45.0)		27 (20.6)	104 (79.4)	
Circular	78 (42.6)	105 (57.4)	** < 0.001**	23 (12.6)	160 (87.4)	0.156
Histologic type						
Adenocarcinoma	282 (53.4)	246 (46.6)		85 (16.1)	443 (83.9)	
Others	6 (22.2)	21 (77.8)	**0.002**	1 (3.7)	26 (96.3)	0.102

*EVI* extramural venous invasion, *MRF* mesorectal fascia, *MRI* magnetic resonance imaging

*Missing values: Clinical N category *n* = 4, EVI *n* = 78, distance from MRF *n* = 296, ulceration *n* = 76, extension in the rectum *n* = 77

The year of treatment initiation showed a significant improvement in downstaging (Table 5). Carcinomas from patients treated between 2011 and 2019 were diagnosed more frequently as ≤ ypT2 than previously treated patients (56% vs 40 and 54%; *p* = 0.025).

**Table 5:** Potential treatment-related predictors of downstaging (*n* = 555)

Characteristics	≤ ypT2	ypT3-4	*p*	ypT0	ypT1-4	*p*
	*n* (%)	*n* (%)		*n* (%)	*n* (%)	
	288 (51.9)	267 (48.1)		86 (15.5)	469 (84.5)	
Start of treatment (year)						
1995–2002	43 (40.2)	64 (59.8)		8 (7.5)	99 (92.5)	
2003–2010	129 (54.0)	110 (46.0)		40 (16.7)	199 (83.3)	
2011–2019	116 (55.5)	93 (44.5)	**0.025**	38 (18.2)	171 (81.8)	**0.035**
Institution of nCRT						
Erlangen	221 (50.8)	214 (49.2)		65 (14.79)	370 (85.1)	
Others	67 (55.8)	53 (44.2)	0.329	21 (17.5)	99 (82.5)	0.493
Chemotherapy regimen*						
5-FU/Capecitabine	120 (49.4)	123 (50.6)		31 (12.8)	212 (87.2)	
Oxaliplatin-based	155 (53.3)	136 (46.7)		53 (18.2)	238 (81.8)	
Irinotecan-based	12 (75.0)	4 (25.0)	0.119	2 (12.5)	14 (87.5)	0.244
Hyperthermia*						
Yes	55 (57.9)	40 (42.1)		19 (20.0)	76 (80.0)	
No	231 (50.5)	226 (49.5)	0.192	66 (14.4)	391 (85.6)	0.172
Interruption, dose reduction, discontinuation*						
No	256 (51.9)	237 (48.1)		78 (15.8)	415 (84.2)	
Yes	27 (49.1	28 (50.9)	0.690	7 (12.7)	48 (87.3)	0.548

*nCRT* neoadjuvant chemoradiotherapy, *5-FU* 5-fluorouracil

*Missing values: chemotherapy regimen *n* = 5, hyperthermia *n* = 3; interruption, dose reduction, discontinuation *n* = 7

With regard to pathological complete regression (ypT0), no patient- or tumour-related predictive factors reached the level of significance. However, over the years, the complete regression increased from 7.5 to 18.2% (*p* = 0.035). Thus, the year of treatment was confirmed as a significant predictive (treatment-related) factor.

Potential blood value-related predictive factors for downstaging to ≤ pT2 or ypT0 are listed in Supplementary Table 1. Elevated neutrophil granulocytes were significantly less frequently associated with a pathological complete response.

In the multivariate logistic regression analysis, the factors cT category, BMI, ECOG score, CEA, histological type, extension in the rectum and the year of the start of the treatment were found to be independent factors for predicting downstaging to ≤ ypT2. In addition, the year of treatment initiation remained an independent significant predictor for pathological complete regression (ypT0; Table 6).

**Table 6:** Multivariate logistic regression: downstaging from cT3-4 to ≤ ypT2/ypT0 (*n* = 341)

**Characteristics**		**Downstaging from cT3-4 to ≤ ypT2**			**Downstaging from cT3-4 to ypT0**		
	***n***	**Odds ratio**	**95%CI**	***p***	**Odds ratio**	**95%CI**	***p***
Clinical T category							
cT3	264	1.0			1.0		
cT4	77	0.5	0.3–0.9	**0.020**	0.7	0.3–1.7	0.480
BMI (kg/m^2^)							
< 18.5	8	0.1	0.0–0.6	**0.015**	0.5	0.1–4.9	0.585
18.5–24.9	127	1.0			1.0		
25.0–29.9	141	0.6	0.4–1.1	0.085	1.0	0.5–2.0	0.949
≥ 30.0	65	0.4	0.2–0.8	**0.011**	0.9	0.4–2.2	0.867
ECOG score*							
0–1	312	1.0			1.0		
2–3	29	0.2	0.1–0.6	**0.003**	0.3	0.1–1.4	0.136
CEA (ng/ml)*							
< 5	234	1.0			1.0		
≥ 5	107	0.4	0.3–0.7	**0.002**	0.7	0.4–1.4	0.331
CRP (mg/l)*							
Norm < 5	187	1.0			1.0		
5– < 10	74	1.1	0.6–2.1	0.684	1.2	0.6–2.4	0.625
10– < 50	68	0.8	0.4–1.6	0.567	0.5	0.2–1.2	0.126
≥ 50	12	1.7	0.4–6.7	0.477	2.3	0.5–11.1	0.304
Histologic type							
Adenocarcinoma	326	1.0			1.0		
Others	15	0.2	0.0–0.8	**0.023**	0.4	0.0–3.0	0.347
Extension in the rectum (clinical)*							
Insular	104	1.0			1.0		
Semicircular	98	0.6	0.3–1.2	0.139	1.1	0.5–2.2	0.885
Circular	139	0.4	0.2–0.7	**0.002**	0.6	0.3–1.2	0.145
Start of treatment (year)							
1995–2002	52	1.0			1.0		
2003–2010	140	1.9	0.9–4.0	0.089	7.2	1.6–32.6	**0.011**
2011–2019	149	1.7	0.8–3.5	0.149	7.7	1.7–34.4	**0.008**

*BMI* body mass index, *ECOG* Eastern Cooperative Oncology Group, *CEA* carcinoembryonic antigen, *CRP* C reactive protein

*Missing values: ECOG score *n* = 42, CEA *n* = 58, CRP *n* = 119, extension in rectum *n* = 77

### Prognosis

**Fig. 1:**
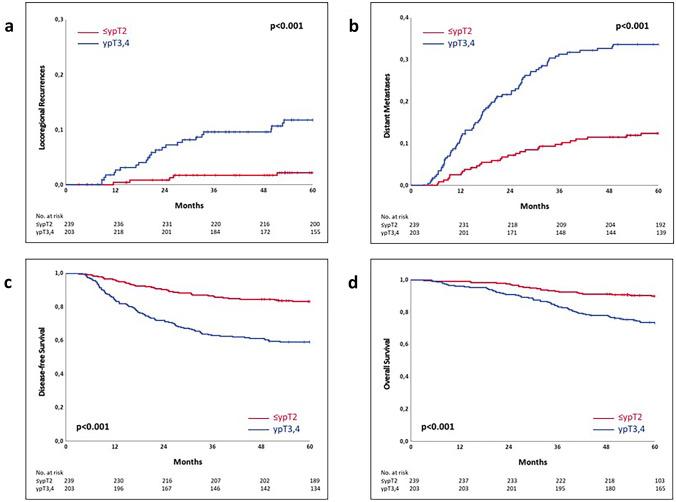
**a** Kaplan–Meier curves of the time to locoregional recurrence (*n* = 471). **b** Kaplan–Meier curves of the time to distant metastases (*n* = 471). **c** Kaplan–Meier curves of disease-free survival (*n* = 471). **d** Kaplan–Meier curves of overall survival (*n* = 471)

For the analysis of prognosis, 239 patients with downstaging to ≤ ypT2 after chemoradiotherapy were compared to 232 patients without downstaging (ypT3-4). In all analyses, for locoregional and distant recurrences as well as for disease-free survival (DFS) and overall survival (OS), the prognosis was superior in patients with downstaging to ≤ ypT2 (*p* < 0.001 each; Table 7, Fig. 1). For locoregional recurrence, the difference was 9 percentage points within 5 years (2.2% vs 11.8%). For distant metastasis, a threefold increase was observed in patients without downstaging (11.9% vs 33.6%). This resulted in a difference of 34 percentage points in DFS (93.2% vs 59.0%) and 16 percentage points in OS (89.9% vs 73.6%).

**Table 7:** Locoregional recurrence, distant metastases, disease-free survival and overall survival after treatment from 1995 to 2015 (*n* = 471)

**Characteristics**	** ≤ ypT2 (*n* = 239)**		**ypT3-4 (*n* = 232)**		
	**5-year rate**	**(SE)**	**5-year rate**	**(SE)**	***p***
Locoregional recurrence	2.2	1.0	11.8	2.2	** < 0.001**
Distant metastases	11.9	2.1	33.6	3.2	** < 0.001**
Disease-free survival	93.2	2.4	59.0	3.2	** < 0.001**
Overall survival	89.9	2.0	73.6	2.9	** < 0.001**
	**ypT0 (*n* = 64)**		**ypT1-4 (*n* = 407)**		
	**5-year rate**	**(SE)**	**5-year rate**	**(SE)**	***p***
Locoregional recurrence	0.0	-	7.9	1.4	**0.014**
Distant metastases	1.6	1.6	26.2	2.2	** < 0.001**
Disease-free survival	96.9	2.2	67.3	2.3	** < 0.001**
Overall survival	98.4	1.6	79.3	2.0	** < 0.001**

This was also evident when comparing the 64 patients with pathological complete regression (ypT0) with the 407 patients diagnosed with ypT1-4. Again, there was a clear difference with a significantly better prognosis in the patients with ypT0. No patient with pathological complete regression had developed locoregional recurrence within 5 years (*p* = 0.014), and only one patient experienced distant metastasis after 26 months (*p* < 0.001). For patients with ypT0, DFS improved by 30 percentage points (96.9% vs 67.3%) and OS by 18 percentage points (98.4% vs 79.3%; *p* < 0.001 each).

## Discussion

Neoadjuvant chemoradiotherapy (nCRT) has significantly improved the prognosis of patients with advanced rectal cancer, in particular by reducing the local recurrence rate. However, there is an ongoing debate about the indication for nCRT, i.e. which patients would actually benefit from nCRT and expect a good response. In this study, we identified several factors in multivariate analysis that may predict downstaging from advanced rectal carcinomas (cT3-4) to ≤ ypT2. We found several patient-related independent predictive factors, such as BMI, ECOG score and CEA; tumour-related predictive factors, such as cT category, extension in the rectum and histological type; and one treatment-related factor reflecting the year of treatment. The latter also proved to be an independent predictive factor for a pathological complete response (ypT0).

### Patient-related predictive factors

In this study, BMI was proven to be an independent predictive factor for downstaging. Normal weight patients experienced significantly better downstaging ≤ ypT2 of the primary tumour than underweight (< 18.5 kg/m^2^) and obese (≥ 30.0 kg/m^2^) patients. This is confirmed by data from Sun et al. and Ottaiano et al., who found that obesity leads to poorer downstaging of the T category and more frequent side effects, which may result in lower doses of chemotherapy [14, 15]. In patients with thoracic tumours, Zhao et al. found an association between increased BMI and raised so-called setup errors at daily positioning for radiation [16].

The ECOG performance score describes the general condition of oncological patients with regard to their functional abilities to care for themselves, their daily activity and ability to work and their need for care [17]. In this study, the ECOG score was identified as an independent predictive factor for downstaging in the multivariate analysis. To our knowledge, this factor has not been investigated in other studies on rectal cancer.

Human carcinoembryonic antigen (CEA) is a broadly used tumour marker recognised for staging, disease surveillance and follow-up. In this study, the pretherapeutic CEA level provides a prediction of the response to nCRT. This is consistent with the results of the studies of Yoon et al. [11], Das et al. [18] and Park et al. [19]. Patients with an elevated CEA level responded worse to nCRT than patients with a regular value. The CEA level was found to be not only a predictive factor for downstaging but also a predictor for pathological complete regression. Ordonez et al. explained the influence of CEA by the fact that CEA inhibits cell apoptosis. Tumour cells that overexpress CEA are therefore resistant to nCRT [20].

### Tumour-related predictive factors

The clinical T category was also confirmed in this study as an independent predictive factor for downstaging ≤ ypT2. This is to be expected, as cT3 carcinomas invade only into the perirectal fat tissue, whereas cT4 carcinomas infiltrate beyond, into visceral peritoneum or neighbouring organs or structures. This is consistent with the results of Yoon et al. [11], who also showed that cT3 is a predictor of advanced tumour downstaging. Zhang et al. [21] demonstrated a strong correlation between the cT category and pathological complete regression of rectal carcinoma. The clinical N category is not used as a benchmark for downstaging because of its low sensitivity and specificity.

Most rectal carcinomas in our study presented as conventional adenocarcinoma (95%). The few mucinous and signet ring carcinomas responded significantly worse to nCRT. This is controversial in the literature. Engineer et al. [22] also described rare downstaging in patients with signet ring cell carcinoma, and McCawley et al. [23] described both a lower rate of downstaging and pathological complete regression in mucinous carcinoma. Jayanand et al. [24] found that the pathological complete regression rate is higher in signet ring cell carcinoma.

The extent of carcinoma in the rectum was identified as another significant predictive factor. An insular noncircular extension resulted in a better response and thus better downstaging. The study by Jayanand et al. [24] also revealed that pathological complete regression was achieved more frequently with noncircumferential extension. This result was also confirmed by Das et al. [18] for both downstaging and pathological complete regression. Subsequently, a circular extension of the carcinoma is associated with a worse prognosis [25, 26].

The distance to the mesorectal fascia on imaging is a relatively new but important factor for indication and prognosis in rectal cancer. As a possible predictive factor, it had to be excluded from our multivariate analysis because of too many missing values. The univariate analysis indicated that it is a predictive factor for downstaging. Ren et al. [27] already proved that the distance of the tumour to the mesorectal fascia significantly influences the chance of pathological complete regression.

### Treatment-related predictive factors

The year of treatment also proved to be a significant factor for downstaging. This could be due to continuous changes and thus improvements in radiation therapy and chemotherapy application. In particular, the use of moderate doses of oxaliplatin led to increased remission, measurable in the frequency of pathological complete remission [28]. In addition, the conversion from 3D radiation techniques to volumetric modulated arc therapy (VMAT) irradiation led to significantly less toxicity of radiation therapy [29]. As a result, it is more often possible to treat patients without an irradiation break, which usually leads to fewer local recurrences [30].

### Prognostic factors

In this study, we identified a variety of predictive factors that are already known as prognostic factors. Predictive factors refer to factors related to response or nonresponse to a specific therapy; in this study, the downstaging of the primary tumour ≤ ypT2. Prognostic factors, on the other hand, are factors related to prognosis, i.e. disease-free survival and overall survival [31, 32].

The anatomical extent of the disease at the start of treatment (TNM) and after surgical treatment (R-classification) has been identified as the most important prognostic factor in solid tumours [33–35]. In this study, the cT category proved to be a powerful independent predictive factor for downstaging and a prognostic factor. Similarly, the histological type and the distance of the tumour from the mesorectal fascia are also important tumour-related prognostic factors in rectal carcinoma.

Among patient-related factors, CEA proved to be a predictive factor and has already been considered a probable essential prognostic factor by the UICC in 2006 [36] and is well supported by the literature [32]. Of particular interest is an elevated BMI, which is first a risk factor for developing colorectal cancer [37–39], second also a predictive factor for downstaging and third a prognostic factor [40–42].

### Nonoperative management (NOM) - watch and wait (W&W) - total neoadjuvant therapy (TNT)

The secondary endpoint of our study was advanced downstaging to ypT0, i.e. pathological complete regression. These patients showed an excellent prognosis with an observed 5-year survival rate of 98.4%. New alternative treatment concepts named ‘nonoperative management’, ‘watch and wait’ or ‘total neoadjuvant therapy’ focus on giving patients with clinically complete remission (without pathological confirmation) only close follow-up without requiring surgery. However, this requires that the endoscopic and radiological assessment of tumour response to nCRT correlates with pathological tumour response[43]. In our department, W&W was not systematically followed. During the long study period from 1995 to 2019, the time interval after nCRT was increasingly extended from 6 to 8 weeks, especially to enable sphincter preservation in individual patients with very low rectal cancer. Finally, in the case of clinical complete remission, a W&W strategy was applied in 36 patients. While 27 patients never required surgery, nine of these patients had to undergo surgical tumour resection after 3 to 41 months.

Patients who do not require surgery are considered to have a better quality of life than patients after low anterior resection and an especially better quality of life than after abdominoperineal excision. However, even after sphincter-preserving surgery, patients may experience a variety of symptoms, such as urgency and frequency of bowel movements, bowel fragmentation, faecal incontinence or abdominal pain, collectively known as ‘low anterior resection syndrome’. Nevertheless, the consequences of chemoradiotherapy must not be disregarded [44].

Another problem is patients in whom complete regression is not achieved despite a longer waiting time after nCRT. After approximately 10 weeks, the optimal time window for surgery has passed. Inflammation, edema and fibrosis of surrounding tissues in the pelvis may have increased over time, potentially resulting in more difficult preparation, prolonged surgery time and higher morbidity [45].

Knowledge of predictive factors for downstaging in rectal cancer may be helpful to offer nCRT/TNT to those patients who are most likely to benefit and to perform primary surgery on those patients who would not benefit from nCRT/TNT.

The strengths of our study are the completeness of the prospectively collected data and a long follow-up time. The limitations of the study refer to the retrospective nature of the study and the single-centre design. In particular, the lack of data on the distance of the tumour from the mesorectal fascia due to the time period chosen limits our study.

## Supplementary Information

Below is the link to the electronic supplementary material.

Supplementary file1 (DOCX 14 KB)
