# Supplementary material for: Factors influencing downstaging after neoadjuvant long-course chemoradiotherapy in rectal carcinoma
Source: Int J Colorectal Dis. 2022 May 11;37(6):1355–65. doi: 10.1007/s00384-022-04174-y (PMC9167202; doi:10.1007/s00384-022-04174-y)
Supplement: Supplementary file 1 — Supplementary file1 (DOCX 14 KB) [file 384_2022_4174_MOESM1_ESM.docx]

**Suppl. Table 1** Potential pretreatment blood value-related predictors of downstaging (n=555)

| **Characteristics** | **≤ypT2** | **ypT3-4** | **p** | **ypT0** | **ypT1-4** | **p** |
| --- | --- | --- | --- | --- | --- | --- |
|  | n (%) | n (%) |  | n (%) | n (%) |  |
|  | 288 (51.9) | 267 (48.1) |  | 86 (15.5) | 469 (84.5) |  |
| Platelets* |  |  |  |  |  |  |
| reduced <160 | 10 (62.5) | 6 (37.5) |  | 3 (18.8) | 13 (81.3) |  |
| Norm | 205 (53.0) | 182 (47.0) |  | 62 (16.0) | 325 (84.0) |  |
| increased >400 | 12 (44.4) | 15 (55.6) | 0.505 | 3 (11.1) | 24 (88.9) | 0.787 |
| Neutrophils* |  |  |  |  |  |  |
| Reduced | 3 (37.5) | 5 (62.5) |  | 3 (37.5) | 5 (62.5) |  |
| Normal | 31 (60.8) | 20 (39.2) |  | 14 (27.5) | 37 (72.5) |  |
| Increased | 62 (54.4) | 52 (45.6) | 0.414 | 17 (14.9) | 97 (85.1) | **0.049** |
| Monocytes |  |  |  |  |  |  |
| Reduced | 0 (0.0) | 3 (100.0) |  | 0 (0.0) | 3 (100.0) |  |
| Normal | 121 (55.0) | 99 (45.0) |  | 45 (20.5) | 175 (79.5) |  |
| Increased | 6 (50.0) | 6 (50.0) | 0.163 | 2 (16.7) | 10 (83.3) | 1.000 |
| Lymphocytes* |  |  |  |  |  |  |
| Reduced | 27 (45.0) | 33 (55.0) |  | 7 (11.17 | 53 (88.3) |  |
| Normal | 145 (54.7) | 120 (45.3) |  | 44 (16.6) | 221 (83.4) |  |
| Increased | 3 (27.3) | 8 (72.7) | 0.104 | 0 (0.0) | 11 (100.0) | 0.303 |

*Missing values: platelets, n=125; neutrophils, n=382; lymphocytes, n=219
